# The Growth Proliferation, Apoptotic Prevention, and Differentiation Induction of the Gelatin Hydrolysates from Three Sources to Human Fetal Osteoblasts (hFOB 1.19 Cells)

**DOI:** 10.3390/molecules23061287

**Published:** 2018-05-28

**Authors:** Ming Lu, Xin-Huai Zhao

**Affiliations:** 1 Key Laboratory of Dairy Science, Ministry of Education, Northeast Agricultural University, Harbin 150030, China; luming8965@163.com; 2 Department of Food Science, Northeast Agricultural University, Harbin 150030, China

**Keywords:** gelatin hydrolysate, human fetal osteoblasts, proliferation, apoptosis, differentiation, Wnt/β-catenin pathway

## Abstract

Gelatins from the skin of bovine, porcine, and tilapia were hydrolyzed to three degrees of hydrolysis (DH) by alcalase, neutrase, and papain, respectively. These hydrolysates at 0.02–0.1 g/L promoted the growth of human fetal osteoblasts by 101.4–135.7%, while higher DH or using papain and tilapia gelatins resulted in higher proliferation. The hydrolysates from porcine and tilapia gelatins at 0.05 g/L prevented induced apoptosis (decreasing total apoptotic proportions from 28.4% or 35.2% to 10.3–17.5% or 16.0–23.6%), and had differentiation induction (increasing alkaline phosphatase activity by 126.9–246.7% in early differentiation stage, or enhancing osteocalcin production by 4.1–22.5% in later differentiation stage). These hydrolysates had a similar amino acid profile; however, tilapia gelatin hydrolysates by papain with DH 15.4% mostly displayed higher activity than others. Tilapia gelatin hydrolysate could up-regulate β-catenin, Wnt 3a, Wnt 10b, cyclin D1, and c-Myc expression at mRNA levels by 1.11–3.60 folds, but down-regulate GSK 3β expression by 0.98 fold. Of note, β-catenin in total cellular and nuclear protein was up-regulated by 1.14–1.16 folds but unchanged in cytoplasmic protein, Wnt 10b, cyclin D1, and c-Myc expression were up-regulated by 1.27–1.95 folds, whilst GSK 3β expression was down-regulated by 0.87 fold. Activation of Wnt/β-catenin pathway is suggested to mediate cell proliferation and differentiation.

## 1. Introduction

Bone as a dynamic living tissue is tightly regulated by two dynamic processes: bone formation and resorption. In general, the osteoclasts create an eroded cavity by removing bone minerals and matrices, whereas the osteoblasts fill the cavity with new bone by producing bone matrices and mineralization [1,2]. The imbalance between bone formation and resorption thus results in the development of osteoporosis, hypercalcemia, and theumatoid arthritis, and other unfavorable events [3,4,5]. As for the osteoporosis, it increases bone fragility and susceptibility to fracture as a result of low bone mass and deterioration of bone micro-architecture [6], and is significantly higher in women after menopause [7]. Clinic medicines using calcium salts, vitamin D, bisphosphonates, estrogen supplement, calcitonin, and other agents provide protective roles against postmenopausal osteoporosis [8]. However, long-term use of these medicines may lead to serious side effects [9], such as increase in breast cancer risk, decrease in body weight, upper gastrointestinal distress, and sciatica [10,11]. In the present time, it is imperative to develop functional foods with positive effects on bone health but without these side effects on the body.

To the present knowledge, soy iso-flavones, milk proteins, and especially collagen hydrolysates have been verified with potential bone benefits [12,13,14]. Collagen hydrolysates show different activities in animal, cell, or clinical experiments. Clinical investigations indicate that collagen hydrolysate can improve joint function in the patients with osteoarthritis [13,15]. Oral administration of hydrolyzed collagen in the animal model leads to enhanced bone mineral density, as well as bone mineral content [16,17]. Intake of shark skin gelatin is able to increase bone mineral density and type I collagen content of the ovariectomized rat femurs [18]. In model cell experiments, a collagen tri-peptide is capable of stimulating in vitro calcification of human osteoblastic cells, and increasing the production and mRNA levels of type I collagen [19], while chum salmon skin gelatin hydrolysates show capacity to accelerate cell proliferation and prevent NaF-induced apoptosis of hFOB 1.19 cells [20]. Moreover, it is also found that the differentiation of osteoblasts can be stimulated by protein hydrolysates [21], resulting in enhanced Runx2 expression, alkaline phosphatase (ALP) activity, and osteocalcin production in MC3T3-E1 cells [22]. However, many animal sources (such as the skin and bone from porcine, bovine, and fish) are available in the present time to produce gelatins. Especially, many commercial proteases such as trypsin, pepsin, alcalase, neutrase, and papain are also available for the production of gelatin hydrolysates. The fact that used gelatin sources and protease types might have a possible influence on the activities of gelatin hydrolysates is important but still not assessed in the present time.

In this study, three gelatin products from the skin of bovine, porcine, and tilapia were hydrolyzed by three proteases (alcalase, neutrase, and papain), respectively, with the aim of generating gelatin hydrolysates with different degrees of hydrolysis (DH). After that, these hydrolysates were used to treat hFOB 1.19 cells and compared for their in vitro activities to the cells including cell proliferation, apoptotic prevention, and differentiation induction. At the same time, some gelatin hydrolysates were assessed for their amino acid compositions. Several genes and proteins of the Wnt/β-catenin pathway were detected for their expression changes in the cells by real-time RT-PCR and Western-blot, to establish the related molecular mechanism.

## 2. Results

### 2.1. Growth Proliferation of Gelatin Hydrolysates for the Osteoblasts

In this study, 26 hydrolysates were prepared from bovine, porcine, and tilapia gelatins using the three proteases and hydrolysis conditions (Table 1). These hydrolysates were named as BAH1–3, BNH1–3, BPH1–2, PAH1–3, PNH1–3, PPH1–3, TAH1–3, TNH1–3, and TPH1–3 (Table 1), with measured DH values close to 7%, 11%, and 15%, respectively. However, the hydrolysate with DH value near to 15% could not be generated using bovine gelatin and papain in this study.­

The positive control 17β-estradiol (E_2_) could promote cell growth (viability values of 121.5–138.6%). When the cells were exposed to these hydrolysates at dose levels of 0.02–0.1 g/L for 24–72 h, the measured values of cell viability were different but all larger than 100% (Table 2). The 26 hydrolysates mostly showed growth acceleration on the cells, resulting in increased viability values as follows: 104.0–123.6% (BAH1–3), 104.1–125.6% (BNH1–3), 104.6–119.5% (BPH1–2), 101.4–124.0% (PAH1–3), 106.1–127.9% (PNH1–3), 102.4–134.4% (PPH1–3), 110.2–135.5% (TAH1–3), 106.3–129.0% (TNH1–3), and 104.3–135.7% (TPH1–3). This fact demonstrates that these hydrolysates had estradiol-like action (but weaker than that of E_2_). Further data comparison indicates that the hydrolysates of higher DH values always had higher activities than the counterpart hydrolysates of lower DH values, and higher cell proliferation was usually achieved at hydrolysate dose of 0.05 g/L. Moreover, cell treatment at 48 h led to the hydrolysates with higher activities. In the most cases, the hydrolysates generated by papain had slightly higher activities than those generated by other proteases, whilst tilapia gelatin hydrolysates were more active than other gelatin hydrolysates. The hydrolysates from bovine gelatin demonstrated the weakest activity to the cells, and therefore were not considered to assess their other activities in this study. Dose level of 0.05 g/L, treatment time of 48 h, and these hydrolysates with DH values of 7% and 15% were used in later evaluation.

### 2.2. Apoptotic Prevention of Gelatin Hydrolysates on the Osteoblasts

**Figure 1:**
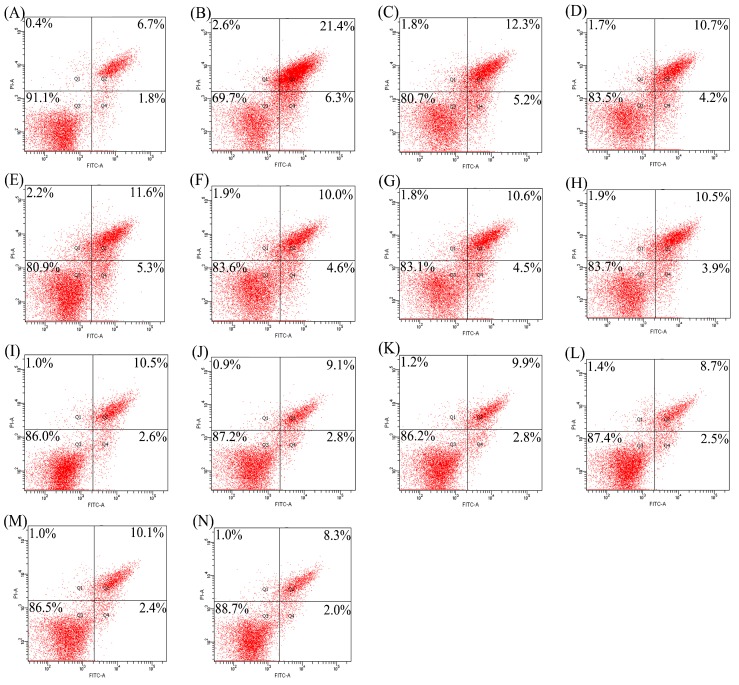
Detection of apoptotic prevention in the osteoblasts by flow cytomety. (**A**) control cells; (**B**) cisplatin-treated cells; (**C**–**H**) the cells firstly treated by respective PAH1, PAH3, PNH1, PNH3, PPH1, and PPH3 for 48 h, and then treated by cisplatin for 24 h; (**I**–**N**) the cells firstly treated by respective TAH1, TAH3, TNH1, TNH3, TPH1, and TPH3 for 48 h, and then treated by cisplatin for 24 h.

**Figure 2:**
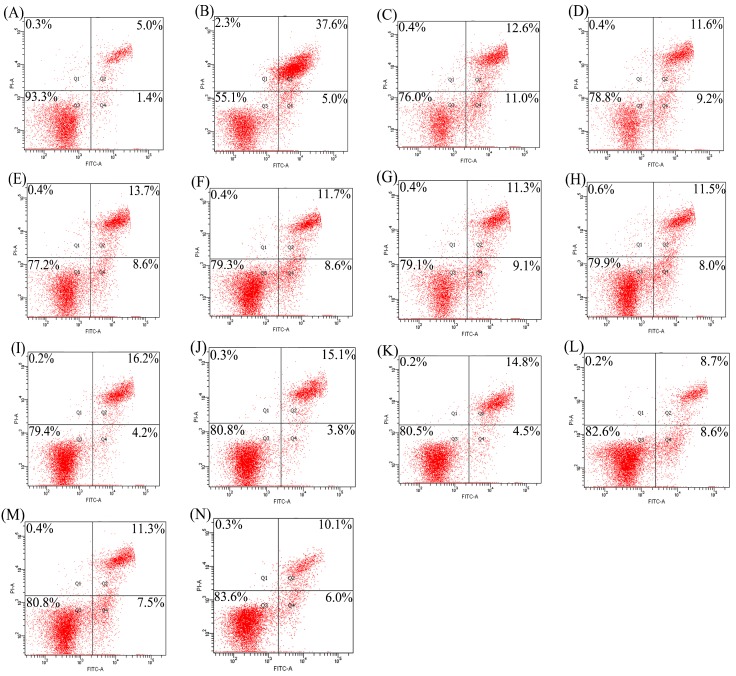
Detection of apoptotic prevention in the osteoblasts by flow cytomety. (**A**) control cells; (**B**) EP-treated cells; (**C**–**H**) the cells firstly treated by respective PAH1, PAH3, PNH1, PNH3, PPH1, and PPH3 for 48 h, and then treated by EP for 24 h; (**I**–**N**) the cells firstly treated by respective TAH1, TAH3, TNH1, TNH3, TPH1, and TPH3 for 48 h, and then treated by EP for 24 h.

**Figure 3:**
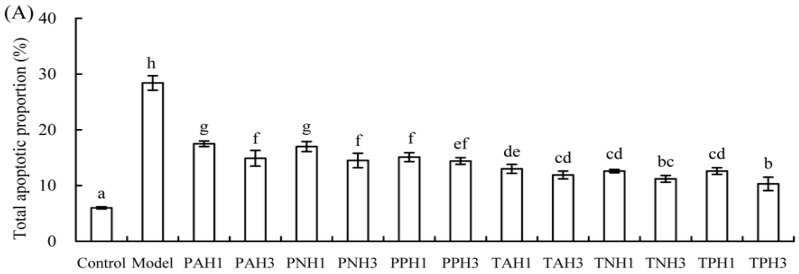

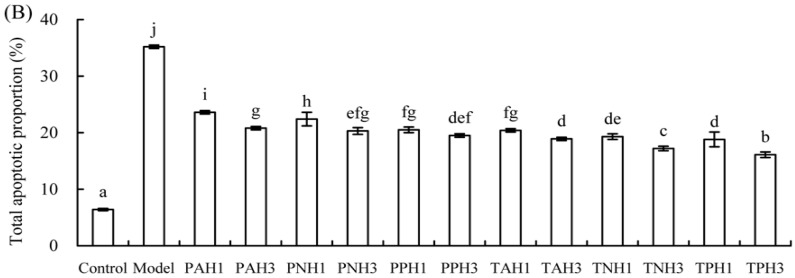
Total apoptotic proportions (%) of the osteoblasts exposed to 12 hydrolysates using cisplatin- (**A**) or EP- (**B**) induced apoptosis. Different letters above the columns indicate that one-way ANOVA of the means differs significantly (*p* < 0.05).

Apoptotic prevention of the hydrolysates was assessed by detecting necrotic (Q1), late apoptotic (Q2), early apoptotic (Q4), and intact (Q3) cell proportions of the treated cells (Figure 1 and Figure 2). Total apoptotic proportions (Q2 + Q4) of the cells treated with different hydrolysates and cisplatin or etoposide (EP) are shown in Figure 3. Total apoptotic proportion of the control cells without any treatment was only 6.5%, while that of model cells using cisplatin or EP treatment showed enhanced total apoptotic proportion of 28.4% or 35.2% (*p* < 0.05). Of note, all hydrolysates displayed apoptotic prevention on the cells by decreasing total apoptotic proportions significantly (*p* < 0.05). They prevented apoptosis of the cisplatin-treated cells, resulting in lower total apoptotic proportions ranging from 10.3% (TPH3) to 17.5% (PAH1). They also antagonized EP-induced apoptosis, as measured total apoptotic proportions ranged from 16.0% (TPH3) to 23.6% (PAH1). Tilapia gelatin hydrolysates always had higher effect than porcine gelatin hydrolysates, while papain-generated hydrolysates consistently exerted better effect than other hydrolysates. It is worth mentioning here that these hydrolysates were more active to antagonize cisplatin-induced apoptosis, because total apoptotic proportions were decreased by 38.3–63.7%; in the case of EP-induced apoptosis, total apoptotic proportions were decreased to 33.0–54.5%.

### 2.3. Differentiation Induction of Gelatin Hydrolysates to the Osteoblasts

**Figure 4:**
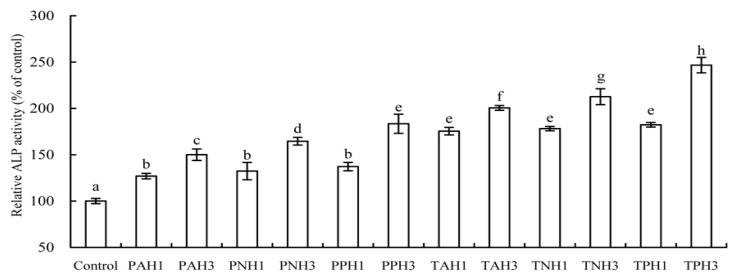
Alkaline phosphatase (ALP) activity of the osteoblasts exposed to 12 gelatin hydrolysates for 7 days. Different letters above the columns indicate that one-way ANOVA of the means differs significantly (*p* < 0.05).

Elevated ALP level indicates early osteoblast differentiation. The results (Figure 4) show that all hydrolysates could enhance ALP activity of the treated cells significantly after a differentiation time of 7 days (*p* < 0.05), as compared to the control cells. Using porcine gelatin hydrolysates resulted in 126.9–183.4% increases in ALP activity, while using tilapia gelatin hydrolysates led to 175.5–246.7% increases in ALP activity. With a brief data comparison, it is found that tilapia gelatin hydrolysates were more effective than porcine gelatin hydrolysates of counterpart protease at inducing osteoblast differentiation (*p* < 0.05), and, mostly, the papain-generated hydrolysates showed higher efficiency than the other hydrolysates (*p* < 0.05).

**Figure 5:**
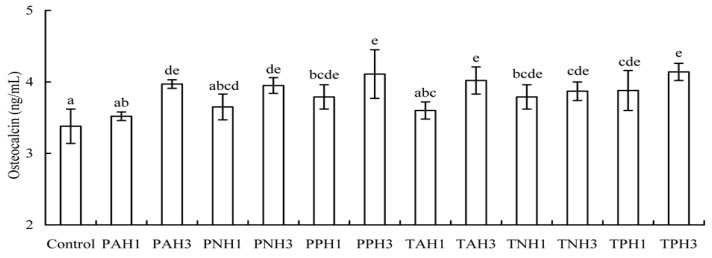
Osteocalcin levels of the osteoblasts exposed to 12 gelatin hydrolysates for 21 days. Different letters above the columns indicate that one-way ANOVA of the means differs significantly (*p* < 0.05).

Osteocalcin is another important index to reflect differentiation extent of the osteoblasts. The results (Figure 5) show that these hydrolysates enhanced osteocalcin content in the cells significantly (*p* < 0.05), as compared to the control cells (3.38 ng/mL). After a differentiation time of 21 days, osteicalcin levels were enhanced to 3.52–4.11 and 3.60–4.14 ng/mL when using porcine gelatin hydrolysates and tilapia gelatin hydrolysates, respectively. That is, osteocalcin production was totally increased by 4.1–22.5%. Data comparison also indicates that tilapia gelatin hydrolysates had stronger differentiation induction than porcine gelatin hydrolysates, and the papain-generated hydrolysates always exerted higher differentiation than other hydrolysates.

**Figure 6:**
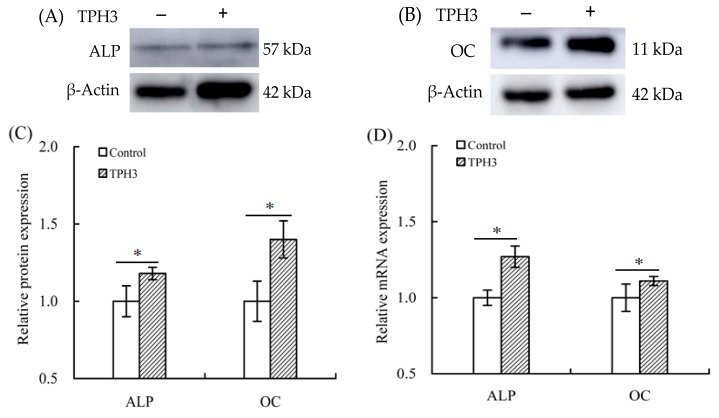
Expression changes of alkaline phosphatase (ALP) and osteocalcin (OC) in the osteoblasts exposed to TPH3. The cells were treated with vehicle or TPH3 (0.05 g/L) for 7 (**A**) and 21 (**B**) d, respectively. Relative protein expression of ALP and OC (**A**,**B**); protein expression changes of ALP and OC (**C**), and relative mRNA expression changes of ALP and OC (**D**). Expression of genes and proteins were normalized against β-actin. *: compared to control, *p* < 0.05.

When TPH3 was used to treat the cells for 7 days and 21 days, it was found that gene expression of ALP and osteocalcin was up-regulated by 1.27- and 1.19-folds, respectively (Figure 6D). At the same time, western-blot assay results also showed that protein expression of ALP and osteocalcin was increased by 1.18- and 1.40-folds, respectively (Figure 6A–C). It is thus proved that TPH3 up-regulated ALP and osteocalcin exprssion at both mRNA and protein levels to promote osteoblast differentiation.

### 2.4. Amino Acid Compositions of Gelatin Hydrolysates

Nine hydrolysates from three gelatins with higher growth proliferation were measured for their amino acid compositions. The results (Table 3) indicate that these hydrolysates in total had slight differences in the content of the measured 18 amino acids; however, their amino acid profiles were very similar. For example, Ala, Arg, Gly, 4-hydroxyproline (4-Hyp), and Pro accounted for a large proportion of total amino acids. Similar amino acid profiles might cause these hydrolysates with similar activity to the osteoblasts. 4-Hyp is a specific amino acid in collagen and its derivatives (e.g., gelatins). Tilapia gelatin hydrolysates had slightly lower 4-Hyp content than the hydrolysates of other sources. Moreover, the used proteases also showed unclear effect on amino acid profiles of the resultant hydrolysates.

### 2.5. The Changes of Gene Expression in the Treated Osteoblasts

**Figure 7:**
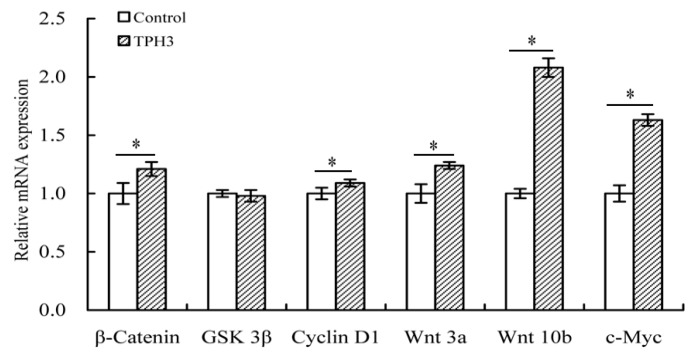
Expression changes of the genes in the osteoblasts. The cells were exposed to vehicle or TPH3 (0.05 g/L) for 48 h. Gene expression was normalized against β-actin. *: compared to control, *p* < 0.05.

After the osteoblasts were treated with TPH3, mRNA expression of several genes related to the Wnt/β-catenin pathway was analyzed. The results (Figure 7) show that TPH3 could up-regulate Wnt 3a, Wnt 10b, β-catenin, cyclin D1, and c-Myc at mRNA levels by respective 1.76-, 2.08-, 1.21-, 1.09-, and 1.63-folds. However, mRNA expression of GSK 3β was slightly down-regulated (by 0.98-fold). That is, TPH3 could regulate osteoblast proliferation via the Wnt/β-catenin pathway.

### 2.6. The Changes of Proteins Expression in the Treated Osteoblasts

**Figure 8:**
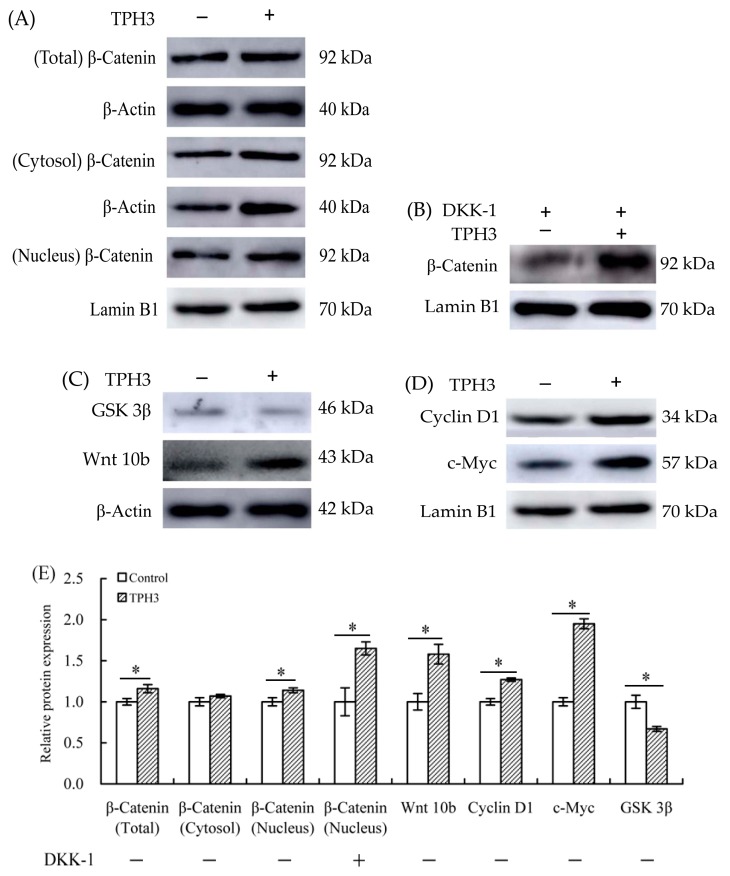
Expression changes of the proteins related to Wnt/β-catenin pathway in the osteoblasts. The cells were exposed to vehicle, TPH3 (0.05 g/L) for 48 h (**A**,**C**,**D**), and DKK-1 (100 ng/mL) in the absence or presence of TPH3 (0.05 g/L) for 48 h (**B**). Relative protein expression was normalized against β-actin in total cellular or cytoplasmic protein, or against Lamin B1 in nuclear protein (**E**). *: compared to control, *p* < 0.05.

Βeta-catenin is the core component of Wnt/β-catenin pathway. After treatment of osteoblasts with TPH3, protein expression of β-catenin was regulated. The expression of β-catenin in total cellular or nuclear protein was increased by 1.16- or 1.14-folds, while that in cytoplasmic protein was unchanged (Figure 8A). TPH3 thus is proved able to activate the Wnt/β-catenin pathway via promoting protein expression of β-catenin in the cells. TPH3 also could promote β-catenin transfer into nucleus and accumulate in nucleus. In the Wnt/β-catenin pathway, both GSK 3β and Wnt 10b are up-stream regulatory proteins, while both cyclin D1 and c-Myc are important down-stream proteins. As seen from Figure 8C, protein expression of GSK 3β was down-regulated by 0.87-fold, while that of Wnt 10b was up-regulated by 1.58-fold. At the same time, proteins expression of cyclin D1 and c-Myc was up-regulated by 1.27- and 1.95-folds (Figure 8D). Of note, when the cells were co-cultured with DKK-1 (potent antagonist of the Wnt/β-catenin signaling) and TPH3, expression level of β-catenin in nuclear protein was higher compared with that cells exposed to DKK-1 alone (Figure 8B). It is concluded from these results that TPH3 promoted osteoblast proliferation via the Wnt/β-catenin pathway, which supports the assaying results of mRNA expression.

## 3. Discussion

Gelatin and gelatin hydrolysates in many studies have been assessed for their effects on bone metabolism in vivo or in vitro. Oral ingestion of gelatin can cause increased bone mineral density [23]. Collagen hydrolysates are able to increase osteoblast activity in vivo or in vitro [24]. Bovine collagen peptides can increase MC3T3-E1 cell proliferation and enhance percentage of the cells in G_2_/S phase by 31.82% [22]. Porcine skin gelatin hydrolysate at 100 ng/mL can stimulate proliferation of MG63 cells by 120% [25]. Chum salmon skin gelatin hydrolysates can promote osteoblast growth by 127–136% [20]. In this study, the assessed gelatin hydrolysates showed growth proliferation and apoptotic prevention on the osteoblasts (Table 2, Figure 1, Figure 2 and Figure 3), demonstrating result consistence with these mentioned studies. However, biological activity of collagen, gelatin, or their hydrolysates is different, depending on collagen types or collagen sources [26]. It is thus reasonable that these assessed hydrolysates were generated from three gelatin products, and thus had different activity values.

Apoptosis or programmed cell death is an essential mechanism by which organisms remove unwanted cells to precisely control organ development and function [27]. Apoptosis is also a crucial determinant of the life span of the osteoblasts in bone-forming function [28]. Estrogen, acting via estrogen receptor α, can promote osteoclast apoptosis [29] but prevent osteoblast apoptosis [30]. Furthermore, estrogen receptor can stimulate a Src/Shc/ERK signaling pathway that prevents apoptosis induced by dexamethasone, EP, and other pro-apoptotic agents [31]. Several protein hydrolysates such as that from chum salmon skin gelatin have been verified with apoptotic prevention, decreasing total apoptotic proportion from 23.6–32.9% to 14.3–15.2% in NaF- or EP-induced apoptosis [32]. In this study, both cisplatin- and EP-induced osteoblast apoptosis were conducted, while the assessed hydrolysates all had apoptotic prevention on the osteoblasts via decreasing total apoptotic proportions significantly (*p* < 0.05, Figure 1, Figure 2 and Figure 3). However, possible molecular mechanisms responsible for anti-apoptotic effect of these gelatin hydrolysates remain unclear in the present time, and should be clarified in future.

ALP is highly expressed at early differentiation of the osteoblasts, and thus served as an early differentiation marker [33]. Moreover, osteocalcin exhibits an important role at later differentiation of the osteoblasts and served as a late differentiation marker [34]. ALP can hydrolyze pyrophosphate and provide inorganic phosphate for the osteoblasts to promote its mineralization [35]. Osteocalcin is the most abundant non-collagenous protein in bone matrix, while carboxylated osteocalcin can bind with hydroxyapatite, resulting in osteocalcin deposition in the extracellular bone matrix [36]. Collagen peptides can increase both ALP activity and osteocalcin level in MC3T3-E1 [22], or enhance ALP activity of rat calvarial cells to 227.57 U/mg protein [34]. Blue mussel (*Mytilus edulis*) protein hydrolysates are verified as being able to stimulate osteoblast differentiation via enhancing ALP activity and osteocalcin production [21]. In total, collagen hydrolysates are able to regulate bone formation and mineralization of bone matrix via stimulating osteoblast differentiation [24]. These mentioned results provide support to the present results; that is, the assessed hydrolysates had differentiation induction via elevating ALP activity in intracellular and increasing osteocalcin production in extracellular (Figure 4, Figure 5 and Figure 6).

Different activities of these hydrolysates in growth proliferation, apoptotic prevention, and differentiation induction might be partially caused by different amino acid compositions and peptide sequences. The lengths and amino acid sequences of peptides are controlled by protease specificity and hydrolysis extent. Hyp in fish skin gelatin hydrolysate is lower than that in porcine skin gelatin hydrolysate [26]. In this study, 4-Hyp also showed relatively lower content in tilapia gelatin hydrolysates of higher activity (Table 3). More importantly, Hyp is a key molecule in collagen hydrolysate intake for the treatment of joint disorders and skin damage [37]. Hyp can inhibit the loss of chondrocytes and thinning of the articular cartilage layer [38], and regulate the differentiation of chondrocytes into their mineralized form [37]. However, the present results did not support that the hydrolysates with relative higher 4-Hyp contents exerted better activities. The assessed hydrolysates in this study with higher DH values always had higher activities, which was consistent with the previous results [20,32,39]. It was mostly found in this study that the papain-generated hydrolysates had higher activities than the osteoblasts (Table 2, Figure 1, Figure 2, Figure 3, Figure 4, Figure 5 and Figure 6). It is thus concluded that protease specificity might make contribution to these activities. How amino acid profile and protease specificity governed hydrolysate activities was out of the scope of this study; however, it should be investigated in future.

Βeta-catenin is a pivotal signaling molecule of the Wnt/β-catenin pathway. Wnt ligands can activate Wnt/β-catenin pathway, which in turn increases β-catenin protein level in the osteoblasts. As the result of this increase, β-catenin accumulates in the cytoplasm, migrates into the nucleus, and thereby initiates the expression of down-stream target genes, which finally leads to cell proliferation and differentiation [40]. Up-regulated β-catenin expression in bone results in increased bone deposition [41,42]. Constitutive activation of β-catenin can ensure the osteoblasts with higher proliferation [33]. In this study, down-regulation of GSK 3β and up-regulation of β-catenin (Figure 8) implied that the Wnt/β-catenin pathway was activated by TPH3. Similarly, a previous study also found that activation of the Wnt/β-catenin pathway was induced via down-regulation of GSK 3β by cytoplasmic protein Dvl [43]. Wnt 1, Wnt 3a, and Wnt 10b can stimulate osteoblaste growth by up-regulating β-catenin expression [44], supporting that TPH3 led to up-regulation of Wnt 10b in the osteoblasts (Figure 8). Cyclin D1 and c-Myc are two key proteins triggering cell cycle mechanism, and are down-stream factors of the Wnt/β-catenin pathway [45]. They are proved able to enhance the growth and proliferation of the calvarial and hMSC cells [46,47]. Consistent with these two studies, this study also found up-regulation of cyclin D1 and c-Myc by TPH3 (Figure 8). DKK-1, a classical antagonist of the Wnt/β-catenin pathway, can competitively bind low-density lipoprotein receptor-related protein 5/6 co-receptors (LRP 5/6, a receptor that binds to Wnt ligands), block their interaction with Wnt ligands, and subsequently cause β-catenin degradation [48]. DKK-1 therefore can inhibit osteoblast proliferation by blocking the Wnt/β-catenin pathway [49]. In this study, TPH3 partially reversed the effect of DKK-1 on the cells (Figure 8). It is thus concluded that gelatin hydrolysate could mediate osteoblast proliferation and differentiation by activating the Wnt/β-catenin pathway.

## 4. Materials and Methods

### 4.1. Materials and Reagents

Bovine gelatin and porcine gelatin were bought from Shandong Yixin Biological Co., Ltd. (Binzhou, Shandong, China), while tilapia gelatin was bought from Guangdong Audima Bioengineering Co., Ltd. (Maoming, Guangdong, China). Alcalase and neutrase with measured activities of 210 and 270 kU/g, respectively, were obtained from Nanning Doing Higher Bio-tech Co., Ltd. (Nanning, Guangxi, China). Papain with measured activity 22 kU/g was obtained from Sinopharm Chemical Reagent Co., Ltd. (Shanghai, China). Fetal bovine serum (FBS) was purchased from Wisent Inc. (Montreal, QC, Canada). Following reagents were purchased from Sigma Chemical Co. (St. Louis, MO, USA): activated charcoal, dexamethasone, DMEM:Ham’s F 12 (1:1) medium, EP, G418, E_2_, and β-glycerophosphate. l-Ascorbic acid was purchased from Xilong Chemical Co., Ltd. (Shantou, Guangdong, China). Cell Counting Kit-8 (CCK-8) was bought from Dojindo Molecular Technologies, Inc. (Kyushu, Japan). The kits used for Annexin V-FITC/PI apoptosis detection, alkaline phosphatase assay, and bicinchoninic acid protein assay, and nuclear or cytoplasmic protein extraction were purchased from Beyotime Institute of Biotechnology (Shanghai, China). RNAprep pure Cell/Bacteria kit was bought from Tiangen Biotech (Beijing, China) Co., Ltd. (Beijing, China). Human Gla-Osteocalcin High Sensitive EIA Kit, PrimeScript^TM^ RT reagent Kit, and SYBR^®^ Rremix Ex Taq™ (Tli RNaseH Plus) all were bought from Takara Bio Inc. (Kusatsu, Japan). Cisplatin was bought from QiLu Pharmaceutical Co., Ltd. (Jinan, Shandong, China). Trypsin-EDTA was bought from Thermo Fisher Scientific Inc. (Grand Island, NY, USA). Dextran T-70 and phosphate-buffered saline (PBS) were bought from Solarbio Science and Technology Co., Ltd. (Beijing, China). Human DKK-1 protein, primary antibodies (β-catenin, GSK 3β, Wnt 10b, cyclin D1, c-Myc, ALP, and osteocalcin), and secondary antibodies were purchased from Abcam plc. (Cambridge, UK). The water used in this study was ultrapure water generated from Milli-Q Plus (Millipore Corporation, New York, NY, USA). Other chemicals used were all analytical grade.

To obtain the estrogen-removed FBS (ER-FBS), commercial FBS was treated with activated charcoal/dextran T-70 as previously described [50]. In briefly, 100 mL FBS was added with 25 mg dextran T-70 and 250 mg activated charcoal, incubated at 55 °C for 45 min, cooled, and centrifuged at 9000× *g* for 15 min at 4 °C. The above steps were repeated three times. After then, the FBS was sterilized by filtration through a 0.22 μm membrane.

### 4.2. Preparation of the Hydrolysates

Three gelatins of 5 g on dry basis were dissolved separately in 100 mL water, adjusted to original pH 8.5, 7.0, and 6.0 with 1 mol/L HCl or NaOH, respectively, and then hydrolyzed at 40 °C using the conditions given in Table 1. After hydrolysis, the solutions were heated for 15 min at 95 °C to terminate the proteases, cooled to 20 °C, and centrifuged at 11,000× *g* for 20 min. The supernatants (hydrolysates) were collected and measured for their DHs. All prepared hydrolysates were freeze-dried with a laboratory freeze dryer (ALPHA 1-4 LSCplus, Marin Christ, Osterode, Germany) and stored at −20 °C for later use.

### 4.3. Chemical Analyses

Nitrogen contents of the samples were measured using the Kjildahl method [51] and a conversion factor of 5.55 to calculate protein contents. Seventeen amino acids of the samples were evaluated by Heilongjiang Provincial Academy of Agricultural Sciences (Harbin, Heilongjiang, China) using an automated amino acid analyzer (L-8800, Hitachi Co., Ltd., Tokyo, Japan) and instrument protocol. 4-Hyp was measured using a reported method [52]. Contents of these 18 amino acids were all expressed as g/kg protein. Moreover, DH values of the hydrolysates were analyzed and calculated as previously described [20].

### 4.4. Cell Line and Culture Conditions

The conditionally immortalized human fetal osteoblast cell line was provided by Cell Bank of the Chinese Academy of Sciences (Shanghai, China). The cells cultured at permissive temperature (33.5 °C) show a rapid cell proliferation, while those cultured at restrictive temperature (39.5 °C) have little or no proliferation but enhanced differentiation [53]. For the assay of growth proliferation, the cells were maintained in a non-differentiation medium consisting of 0.3 g/L G418 and DMEM:Ham’s F-12 (1:1) without phenol red supplemented with 10% (*v*/*v*) ER-FBS, and then were cultured in a humidified incubator with 95% air and 5% CO_2_ at 33.5 °C. For the assay of differentiation induction, the cells were maintained in the incubator at 39.5 °C in a differentiation medium (i.e., the non-differentiation medium supplemented with respective dexamethasone, β-glycerophosphate, and L-ascorbic acid of 0.1 μmol, 10 mmol, and 50 mg per liter) [54].

### 4.5. Measurements of Growth Proliferation and Induced Apoptosis

The cells seeded in a 96-well plate with cell density of 5 × 10^3^ cells per well were incubated at 33.5 °C and starved overnight with the non-differentiation medium consisting of 0.5% (*v*/*v*) ER-FBS. The medium was replaced with 200 μL per well fresh medium containing hydrolysates (0.02–0.1 g/L) or E_2_ (10^−8^ mol/L). The cells were further incubated for 24–72 h before assaying their viability values. After removal of the medium, CCK-8 solution (10 μL CCK-8 in 100 μL non-differentiation medium) of 100 μL was added into each well. The cells were incubated for another 4 h. Finally, OD value of each well was measured at 450 nm by a microplate reader (Bio-Rad Laboratories, Hercules, CA, USA). The cells without gelatin hydrolysates or E_2_ treatment were designed with viability value of 100%.

Two pro-apoptotic chemicals cisplatin and EP were used to induce osteoblast apoptosis, whilst Annexin V-FITC (green fluorescence) and propidium iodide (PI, red fluorescence) were used to discriminate the cells as intact, early apoptotic, late apoptotic, and necrotic cells [55]. The cells seeded in 6-well plates with cell density of 6 × 10^4^ cells per well were grown to about 80% confluence, treated with the non-differentiation medium consisting of hydrolysates (0.05 g/L) for 48 h, and then treated for 24 h with cisplatin (6 mg/L) or EP (10 mg/L). The cells without any treatment were regarded as control group. Then, Annexin V-FITC/PI Apoptosis Detection Kit was used as fellow. The harvested cells were resuspended in 500 µL Annexin V-FITC binding buffer containing 5 µL Annexin V-FITC and 10 µL PI, kept from light at 20 °C for 20 min, and assayed by a flow cytometry (FACS Calibur, Becton Dickson, San Jose, CA, USA) to detect the intact, early apoptotic, late apoptotic, and necrotic cells.

### 4.6. Assays of Alkaline Phosphatase Activity and Osteocalcin Content

The cells seeded in 6-well plates with cell density of 6 × 10^4^ cells per well were grown to 80% confluence with the non-differentiation medium at 33.5 °C and then cultured in the presence or absence of hydrolysates in the differentiation medium for 7 days at 39.5 °C, with regular replacement of fresh medium every 3 day. ALP activity was measured using ALP Assay Kit and the kit instruction. The cells were washed three times by a PBS (10 mmol/L, pH 7.3), incubated with 100 μL lysis buffer on ice for 30 min, and centrifuged at 12,000× *g* for 5 min to collect the supernatants for ALP analysis. OD value of each well was detected by the microplate reader at 405 nm. The cells were corrected by total protein content using the BCA Assay Kit and kit protocol.

The cells seeded in 12-well plates with cell density of 4 × 10^4^ cells per well were cultured in the non-differentiation medium at 33.5 °C to 80% confluence. Afterwards, the medium was replaced with differentiation medium (containing hydrolysates) for 21 days at 39.5 °C. This differentiation medium was also replaced regularly every 3 day. Osteocalcin secreted in cell culture supernatant was measured using the Human Gla-Osteocalcin High Sensitive EIA Kit and kit protocol. OD value of each was also measured by the microplate reader at 450 nm.

### 4.7. Real-Time RT-PCR Analysis

The cells were cultured in the non-differentiation medium containing TPH3 (0.05 g/L) at 33.5 °C for 48 h, or in the differentiation medium containing TPH3 (0.05 g/L) at 39.5 °C for 7 or 21 days. Total RNA was extracted using the RNAprep pure Cell/Bacteria Kit. The cDNA was synthesized from 1 µg of total RNA using the PrimeScript^TM^ RT reagent Kit. SYBR^®^ Rremix Ex Taq^TM^ (Tli RNaseH Plus) was used in real-time PCR, while each real-time PCR was carried out in triplicate for a total 25 μL reaction mixtures at the Applied Biosystems StepOnePlus Real-time PCR System (Life Technologies Corporation, Carlsbad, CA, USA). The mixtures were subjected to 40 cycles of amplification at 95 °C for 30 s, followed by 60 °C for 30 s. The used primers sequences are listed in Table 4. Relative expression of each gene was calculated as 2^−∆∆Ct^ [56], and β-actin expression was calculated as control for normalization.

### 4.8. Western-Blot Assay

After the culture medium was removed, cell layers were washed three times by pre-cooled PBS. The cells were collected by scraping and lysed with RIPA cell lysate buffer supplemented with PMSF (1 mmol/L) on ice for 30 min. The lysate was centrifuged at 14,000× *g* for 5 min at 4 °C. The supernatant was collected as total cellular protein. Both cytoplasmic and nuclear proteins were carried out according to the instructions of the Nuclear and Cytoplasmic Protein Extraction Kit. Protein concentration was determined by the BCA Protein Assay Kit. Protein (50 mg) from each sample was loaded on 10–15% SDS-PAGE and transblotted to a PVDF membrane. The blots were blocked in blocking buffer (5% fat-free powdered milk/0.1% Tween 20 in 10 mmol/L PBS) for 2 h at 37 °C and probed with a 1:1000–1:10,000 dilution of primary antibody in blocking buffer for 1.5 h at 37 °C. Subsequently, the bands were incubated with anti-mouse secondary antibody horseradish peroxidase conjugatem. The enhanced chemiluminescence (ECL) was covered on the PVDF membrane; after then, an ImageQuant LAS 500 chemiluminescence CCD camera (GE Healthcare UK Limited, London, UK) was used to detect the signal. As usual, protein expression in total cellular and cytoplasmic proteins was normalized against β-actin [35], whilst that in nuclear protein was normalized against Lamin B1 as recommended [57]. Finally, the intensity of protein band was calculated by ImageJ software (National Institutes of Health, Bethesda, MD, USA).

### 4.9. Statistical Analysis

All reported date were expressed as means or means ± standard deviations from three independent preparations or evaluations. Statistical analysis was performed by using the SPSS software version 16.0 (SPSS Inc., Chicago, IL, USA) using one-way ANOVA with Duncan’s multiple range tests. The statistical significance was set at a level of *p* < 0.05.

## 5. Conclusions

When the three gelatin products were hydrolyzed by three proteases, the resultant hydrolysates all demonstrated activities (but different potentials) to the assessed osteoblasts, leading to enhanced growth proliferation, apoptotic prevention, and differentiation induction. Overall, papain-induced hydrolysis and higher hydrolysis extent led to higher activity, while the hydrolysates generated from tilapia gelatin and bovine gelatin showed respective higher and lower growth proliferation. The hydrolysates also could prevent cisplatin/EP-induced osteoblast apoptosis, and increase both intracellular ALP activity and extracellular osteocalcin production to induce osteoblast differentiation. Amino acid profile of the hydrolysates and specificity of the proteases might contribute different activities of these hydrolysates. Of note, activation of the Wnt/β-catenin pathway is suggested responsible for the promoted osteoblast proliferation and differentiation of gelatin hydrolysates, via assaying mRNA and protein expression changes of the osteoblasts.

## Figures and Tables

**Table 1:** Preparation conditions and measured degrees of hydrolysis (DH).

Hydrolysates	Hydrolysis Times (h)	Proteases and Used Levels	DH (%)
BAH1	0.5	Alcalase, 5.5 kU/g protein	7.1
BAH2	3.5		11.2
BAH3	13		14.6
BNH1	0.5	Neutrase, 6.0 kU/g protein	6.7
BNH2	2		10.7
BNH3	11		14.8
BPH1	1	Papain, 1.5 kU/g protein	7.3
BPH2	15		10.8
PAH1	0.25	Alcalase, 7.0 kU/g protein	6.5
PAH2	3		11.1
PAH3	12.5		15.2
PNH1	0.3	Neutrase, 6.0 kU/g protein	6.5
PNH2	3		10.8
PNH3	12.5		15.2
PPH1	0.25	Papain, 1.5 kU/g protein	7.2
PPH2	3		11.0
PPH3	12.5		15.0
TAH1	0.3	Alcalase, 6.0 kU/g protein	6.8
TAH2	3		11.2
TAH3	13		14.6
TNH1	0.5	Neutrase, 6.0 kU/g protein	7.3
TNH2	2		10.9
TNH3	12		14.8
TPH1	0.25	Papain, 1.5 kU/g protein	7.2
TPH2	1.5		10.5
TPH3	7		15.4

BAH, BNH, and BPH (or PAH, PNH, and PPH, or TAH, TNH, and TPH) represent the hydrolysates generated from bovine (or porcine, or tilapia) gelatin by alcalase, neutrase, and papain, respectively.

**Table 2:** Cell viability (%) of the osteoblasts treated with different hydrolysates for 24, 48 or 72 h.

Samples	Hydrolysate Doses and Treating Times of the Cells								
	0.02 g/L			0.05 g/L			0.1 g/L		
	24 h	48 h	72 h	24 h	48 h	72 h	24 h	48 h	72 h
BAH1	107.2 ± 4.8^abcd^	112.4 ± 4.8^abcdefg^	107.9 ± 4.0^abcdef^	115.8 ± 1.9^abcde^	117.0 ± 4.6^ab^	113.9 ± 2.7^abcd^	104.0 ± 3.3^a^	109.9 ± 5.7^abc^	112.6 ± 3.3^defghij^
BAH2	108.6 ± 2.0^abcd^	112.7 ± 5.3^abcdefg^	108.1 ± 3.0^abcdef^	119.8 ± 6.8^abcde^	118.2 ± 3.2^abc^	118.3 ± 4.0^abcdefg^	104.5 ± 5.7^ab^	107.6 ± 5.4^a^	117.6 ± 4.6^ghij^
BAH3	116.8 ± 4.4^ef^	119.5 ± 3.4^efg^	113.5 ± 3.4^fghij^	122.5 ± 3.6^cde^	123.6 ± 4.3^abcde^	119.3 ± 2.0^cdefg^	114.7 ± 4.2^defg^	114.2 ± 2.4^abcdefg^	115.0 ± 1.6^efghij^
BNH1	107.7 ± 4.6^abcd^	108.7 ± 4.4^ab^	105.7 ± 5.7^abcd^	114.9 ± 4.4^abc^	116.9 ± 5.9^ab^	111.3 ± 5.1^a^	107.2 ± 4.9^abcd^	108.5 ± 3.7^ab^	107.8 ± 3.6^abcde^
BNH2	108.9 ± 2.6^abcde^	112.1 ± 3.6^abcdef^	108.3 ± 3.7^abcdef^	115.5 ± 1.0^abcd^	118.6 ± 1.7^abc^	115.3 ± 5.1^abcde^	104.1 ± 5.4^a^	110.6 ± 4.0^abc^	107.5 ± 5.1^abcd^
BNH3	111.0 ± 1.9^bcdef^	119.3 ± 5.4^defg^	109.4 ± 1.8^bcdefg^	117.2 ± 2.2^abcde^	125.6 ± 5.2^cde^	117.2 ± 4.0^abcdef^	108.3 ± 4.0^abcde^	115.2 ± 2.6^abcdefg^	108.7 ± 3.3^abcde^
BPH1	104.6 ± 4.3^abc^	109.3 ± 6.2^abc^	109.6 ± 2.1^bcdefg^	114.4 ± 3.4^abc^	116.5 ± 2.7^a^	114.8 ± 3.0^abcd^	108.6 ± 2.6^abcde^	113.1 ± 2.6^abcde^	110.7 ± 1.6^bcdefg^
BPH2	106.3 ± 2.6^abcd^	115.0 ± 2.5^abc^	111.7 ± 2.0^defghi^	116.1 ± 1.7^abcde^	122.8 ± 4.6^abcde^	119.5 ± 2.3^cdefg^	109.2 ± 4.6^abcde^	110.7 ± 2.6^abc^	112.4 ± 2.9^defghi^
PAH1	101.4 ± 2.9^a^	108.8 ± 3.3^abc^	102.4 ± 1.9^a^	113.4 ± 5.8^ab^	120.4 ± 3.5^abcd^	111.7 ± 3.4^ab^	104.6 ± 1.4^ab^	110.6 ± 3.5^abc^	104.9 ± 2.7^abc^
PAH2	103.5 ± 5.3^ab^	109.7 ± 5.6^abc^	103.7 ± 2.4^ab^	119.9 ± 4.2^abcde^	123.3 ± 4.7^abcde^	112.9 ± 5.0^abc^	106.3 ± 2.4^abc^	114.9 ± 4.8^abcdefg^	104.4 ± 4.8^ab^
PAH3	107.4 ± 4.6^abcd^	105.4 ± 1.9^a^	107.5 ± 3.1^abcdef^	122.7 ± 4.3^cde^	124.0 ± 3.6^abcde^	115.9 ± 5.4^abcdef^	114.8 ± 4.5^defg^	115.1 ± 5.5^abcdefg^	111.4 ± 5.8^bcdefgh^
PNH1	106.1 ± 4.1^abcd^	110.7 ± 3.0^abcd^	109.7 ± 1.4^bcdefg^	115.7 ± 4.6^abcde^	121.9 ± 4.3^abcde^	113.3 ± 5.4^abc^	112.4 ± 1.7^bcdefg^	111.8 ± 5.4^abcd^	116.1 ± 3.4^fghij^
PNH2	111.6 ± 4.6^bcdef^	116.5 ± 5.2^bcdefg^	111.6 ± 3.5^defgh^	121.8 ± 2.4^cde^	124.9 ± 5.3^bcde^	117.9 ± 3.4^abcdefg^	117.1 ± 3.5^fg^	121.0 ± 2.6^efghi^	108.7 ± 2.3^abcde^
PNH3	112.4 ± 4.6^cdef^	119.7 ± 4.9^efg^	114.9 ± 2.9^ghij^	124.0 ± 4.1^e^	127.9 ± 2.4^def^	118.9 ± 2.2^bcdefg^	109.4 ± 4.3^abcde^	116.7 ± 4.8^bcdefg^	111.8 ± 5.0^cdefgh^
PPH1	107.6 ± 5.9^abcd^	115.5 ± 5.6^bcdefg^	106.9 ± 4.7^abcdef^	115.6 ± 5.1^abcd^	122.9 ± 3.3^abcde^	115.3 ± 4.5^abcde^	109.5 ± 3.0^abcde^	113.3 ± 3.4^abcdef^	102.4 ± 4.8^a^
PPH2	111.8 ± 5.9^bcdef^	117.4 ± 4.4^cdefg^	110.4 ± 4.0^cdefgh^	118.5 ± 5.1^abcde^	126.8 ± 2.6^de^	120.2 ± 3.6^cdefg^	112.8 ± 4.3^cdefg^	117.9 ± 5.2^cdefgh^	113.2 ± 2.9^defghij^
PPH3	113.7 ± 4.6^def^	120.3 ± 4.6^fg^	116.4 ± 2.8^hij^	119.0 ± 5.4^abcde^	134.4 ± 4.3^fg^	124.9 ± 2.3^g^	116.1 ± 5.0^efg^	128.5 ± 5.1^i^	119.6 ± 4.4^j^
TAH1	111.2 ± 5.3^bcdef^	112.0 ± 5.9^abcdef^	109.4 ± 2.9^bcdefg^	114.4 ± 3.3^abc^	121.8 ± 4.7^abcde^	113.0 ± 5.3^abc^	111.6 ± 3.5^abcdef^	119.6 ± 2.3^defgh^	110.7 ± 2.3^bcdefg^
TAH2	111.9 ± 4.1^bcdef^	113.5 ± 3.6^abcdefg^	111.3 ± 4.3^defgh^	121.2 ± 1.5^bcde^	123.7 ± 3.5^abcde^	116.0 ± 1.0^abcdef^	110.2 ± 3.3^abcdef^	116.1 ± 5.5^abcdefg^	110.9 ± 3.1^bcdefg^
TAH3	118.3 ± 2.6^f^	119.9 ± 3.7^efg^	118.2 ± 5.3^ij^	122.0 ± 4.5^cde^	135.5 ± 3.9^g^	121.3 ± 2.8^defg^	119.7 ± 2.1^g^	121.6 ± 2.7^fghi^	118.6 ± 3.2^hij^
TNH1	106.5 ± 4.4^abcd^	112.9 ± 2.1^abcdefg^	106.3 ± 3.8^abcde^	111.8 ± 3.9^a^	122.6 ± 4.2^abcde^	119.0 ± 4.2^bcdefg^	107.7 ± 5.6^abcd^	115.7 ± 2.9^abcdefg^	112.9 ± 3.0^defghij^
TNH2	106.9 ± 3.9^abcd^	114.3 ± 2.9^bcdefg^	112.9 ± 2.2^efghij^	118.3 ± 5.8^abcde^	125.8 ± 5.0^cde^	122.6 ± 4.7^efg^	108.1 ± 4.3^abcd^	116.6 ± 5.8^bcdefg^	114.0 ± 2.1^defghij^
TNH3	108.9 ± 4.4^abcde^	116.6 ± 4.4^bcdefg^	112.1 ± 2.2^defghi^	123.6 ± 3.2^de^	129.0 ± 4.2^efg^	124.8 ± 3.4^g^	110.2 ± 2.4^abcdef^	122.1 ± 2.1^ghi^	119.0 ± 4.7^ij^
TPH1	108.3 ± 5.3^abcd^	111.4 ± 5.5^abcde^	104.3 ± 4.3^abc^	115.4 ± 4.8^abcd^	122.3 ± 4.3^abcde^	110.9 ± 1.6^a^	110.3 ± 3.9^abcdef^	113.9 ± 6.3^abcdefg^	109.3 ± 3.6^abcdef^
TPH2	107.9 ± 4.6^abcd^	119.2 ± 2.8^defg^	113.4 ± 2.3^fghij^	115.1 ± 5.7^abc^	124.1 ± 3.8^abcde^	123.3 ± 3.7^fg^	106.1 ± 4.9^abc^	119.6 ± 4.6^defgh^	117.0 ± 4.2^ghij^
TPH3	110.7 ± 2.7^bcdef^	120.8 ± 3.7^g^	118.7 ± 2.6^j^	118.4 ± 4.4^abcde^	135.7 ± 2.8^g^	125.3 ± 4.2^g^	109.2 ± 4.6^abcde^	125.4 ± 5.2^hi^	119.2 ± 3.0^ij^

All values shown represent means ± standard deviations of triplicate measurements. Different superscript letters in the same column indicate that one-way ANOVA of the means differs significantly (*p* < 0.05).

**Table 3:** Amino acid compositions (g/kg of protein) of the assessed hydrolysates.

Amino Acids	BAH3	BNH3	BPH2	PAH3	PNH3	PPH3	TAH3	TNH3	TPH3
Ala	98.7	98.9	94.1	100.0	98.6	99.6	103.9	101.6	101.3
Arg	98.7	98.9	94.1	96.4	96.8	95.9	100.4	101.6	97.6
Asp	40.2	39.6	40.0	41.1	48.4	40.6	40.5	35.7	39.8
Cys	7.3	7.2	7.1	7.1	7.2	7.4	7.0	7.1	7.2
Glu	87.8	88.1	89.4	89.3	91.4	88.6	88.0	87.3	86.8
Gly	223.0	226.6	221.2	223.2	222.2	225.1	221.8	228.2	220.6
His	5.5	5.4	4.7	5.4	5.4	5.5	5.3	5.3	5.4
4-Hyp	115.9	118.4	117.9	119.7	120.4	122.6	112.4	113.8	119.3
Ile	14.6	14.4	14.1	12.5	12.5	12.9	14.1	14.3	14.5
Leu	32.9	32.4	32.9	32.1	32.3	33.2	31.7	32.1	30.7
Lys	34.7	34.2	35.3	33.9	35.8	35.1	33.5	33.9	34.4
Met	12.8	12.6	11.8	10.7	10.8	11.1	14.1	14.3	14.5
Phe	18.3	18.0	18.8	17.9	19.7	18.5	19.4	19.6	18.1
Pro	133.5	134.9	131.8	137.5	127.2	136.5	132.0	133.7	130.2
Ser	27.4	25.2	25.9	26.8	30.5	24.0	28.2	23.2	28.9
Thr	7.3	9.0	9.4	10.7	12.5	11.1	10.6	14.3	10.8
Tyr	5.5	5.4	4.7	3.6	3.6	3.7	3.5	3.6	3.6
Val	20.1	21.6	23.5	19.6	21.5	22.1	19.4	19.6	19.9

**Table 4:** The primers used in real-time RT-PCR assays.

Genes	Primers (5′-3′)
Wnt 3a	F-GGTGGCTGTAGCGAGGACAT R-TTGTTGTGGCGGTTCATGGC
Wnt 10b	F-TCCTGACTTCTGTGAGCGAGACC R-CATAGCAGCACCAGTGGAAGCG
β-catenin	F-AGAAGGTCCGAGTGCTGCTC R-CTGAGCTGGCTGTTGACCAC
GSK 3β	F-TTCCTCCTCATGCTCGGATT R-CAGGTGGAGTTGGAAGCTGA
Cyclin D1	F-CAGAAGTGCGAGGAGGAGGT R-TAGAGGCCACGAACATGCAA
c-Myc	F-CGAGGAGAATGTCAAGAGGCGAAC R-GCTTGGACGGACAGGATGTATGC
ALP	F-CGAGTGAACAGGAACAACG R-AATTCTGCCTCCTTCCACCA
Osteocalcin	F-CCAGGCGCTACCTGTATCAA R-GGTCAGCCAACTCGTCACAG
β-Actin	F-CCTGGCACCCAGCACAAT R-GGGCCGGACTCGTCATAC

## Abbreviations

ALP	Alkaline phosphatase
BAH	Bovine skin gelatin hydrolysate by alcalase
BNH	Bovine skin gelatin hydrolysate by neutrase
BPH	Bovine skin gelatin hydrolysate by papain
CCK-8	Cell counting kit-8
DH	Degree of hydrolysis
DKK-1	Dickkopf-1
E_2_	17β-estradiol
EDTA	Ethylenediamine tetra-acetic acid
EP	Etoposide
ER-FBS	Estrogen-removed fetal bovine serum
FBS	Fetal bovine serum
hFOB 1.19 cells	Human fetal osteoblastic cells
PAH	Porcine skin gelatin hydrolysate by alcalcase
PBS	Phosphate-buffered saline
PNH	Porcine skin gelatin hydrolysate by neytrase
PPH	Provine skin gelatin hydrolysate by papain
TAH	Tilapia skin gelatin hydrolysate by alcalase
TNH	Tilapia skin gelatin hydrolysate by neutrase
TPH	Tilapia skin gelatin hydrolysate by papain
